# Association between surgery treatment delays and survival outcomes in patients with esophageal cancer in Hebei, China

**DOI:** 10.3389/fonc.2024.1463517

**Published:** 2024-10-28

**Authors:** Xing Cui, Chunxiao Shi, Xin Chen, Qi Zhao, Jidong Zhao

**Affiliations:** ^1^ Department of Thoracic Surgery, the Fourth Hospital of Hebei Medical University, Shijiazhuang, China; ^2^ Department of Cardiology, the Fourth Hospital of Hebei Medical University, Shijiazhuang, China

**Keywords:** esophageal cancer, delay, overall survival, cancer-specific survival, China

## Abstract

**Introduction:**

The delays in cancer therapies have the potential to impact disease progression by allowing the unchecked growth and spread of cancer cells. However, the understanding of the association between treatment waiting time and survival outcomes in patients with esophageal cancer (EC) is limited. This study aims to assess the impact of waiting time on survival outcomes among EC patients in Hebei province, which is recognized as one of the high-risk areas for EC in China.

**Methods:**

A total of 9,977 non-metastatic EC patients who underwent surgical treatment were identified between 2000 and 2020. The survival outcomes of overall survival (OS) and cancer-specific survival (CSS) were determined using the Kaplan-Meier methodology. Univariate and multivariate Cox regression analysis was employed to evaluate the impact of treatment delays on OS and CSS.

**Results:**

The average delay time for initiating EC surgical treatment after diagnosis was 1.31 months (95%CI=1.29–1.34). Patients with a long delay (≥ 3 months) in treatment, comprising 9977 EC patients, exhibited significantly lower rates of 3-, 5-, and 10-year OS and CSS compared to those without any delay in treatment initiation. A long delay in EC treatment independently associated with an elevated risk of all-cause and cancer-cause mortality among various patient subgroups, including males, older individuals, single individuals, low-income patients, residents of nonmetropolitan counties, as well as those diagnosed with poorly differentiated and stage IV EC.

**Discussion:**

The long delay of treatment initiation impacts the outcomes of OS and CSS in EC patients. Optimizing treatment timing may enhance life expectancy for individuals diagnosed with EC.

## Introduction

Cancer presents a significant public health challenge and plays a pivotal role in the global burden of diseases (1–3). According to the latest data from the Global Cancer Observatory (GLOBOCAN) 2020 database, the impact of cancer on human health is staggering. Approximately 10 million deaths occur worldwide each year due to cancer, which equates to one death every six seconds (4). The incidence of EC ranks eighth globally, while it stands as the sixth leading cause of cancer-related mortality worldwide (5). According to a recent study, the leading causes of cancer deaths in China in 2022 were lung cancer (733,300 deaths), followed by liver cancer (316,500 deaths), stomach cancer (260,400 deaths), colorectal cancer (240,000 deaths), and esophageal cancer (187,500 deaths). These five types of cancers accounted for 67.50% of total cancer deaths in China (6). In 2021, there were approximately 24,318 new cases of esophageal cancer and 18,226 deaths reported in Hebei Province (7).

For cancers that can be screened, efforts have been directed towards the early identification of cancer cases, leading to a higher percentage of patients being diagnosed at an early stage when surgical intervention offers a potential cure. The increasing number of patients diagnosed with malignant cancer will consequently be provided with greater access to medical interventions (8, 9). Unfortunately, the growing complexity of multimodality cancer care led to a significant time gap of several weeks between diagnosis and treatment (10–15). Continuous evidence from systematic reviews and meta-analyses indicate that variations in the effects of extended waiting periods between diagnosis and treatment on clinical outcomes were detected among individuals diagnosed with different forms of cancer (16–18). Several studies demonstrated that the relationship of treatment delays with an increased overall risk of mortality in patients diagnosed with various types of cancers, including but not limited to lung cancer (10, 11), colorectal cancer (12), endometrial cancer (13), liver cancer (14) and female-specific malignancies (15). The available evidence suggested an increase in wait times for EC treatment over the past decade, as well as disparities among different racial populations in the United States (19, 20). Nevertheless, the impact of treatment waiting time on survival outcomes in Chinese patients with EC remains uncertain.

The objective of this study is to investigate whether the duration from diagnosis to surgical treatment has an impact on the OS and CSS among Chinese patients with EC in Hebei, a region identified as one of the high-risk areas for EC. Furthermore, we aimed to assess the association between the time from diagnosis to treatment and the risk of all-cause and cancer-specific mortality.

## Materials and methods

### Ethics statement

Ethical approval in this study was obtained from the Institutional Review Board (IRB) of the Fourth Hospital of Hebei Medical University (IRB2023-101923). Written informed consent was obtained from all the subjects. The study strictly followed the ethical principles delineated in the 1964 Helsinki Declaration along with its subsequent amendments or comparable ethical guidelines.

### Study population

**Figure 1:**
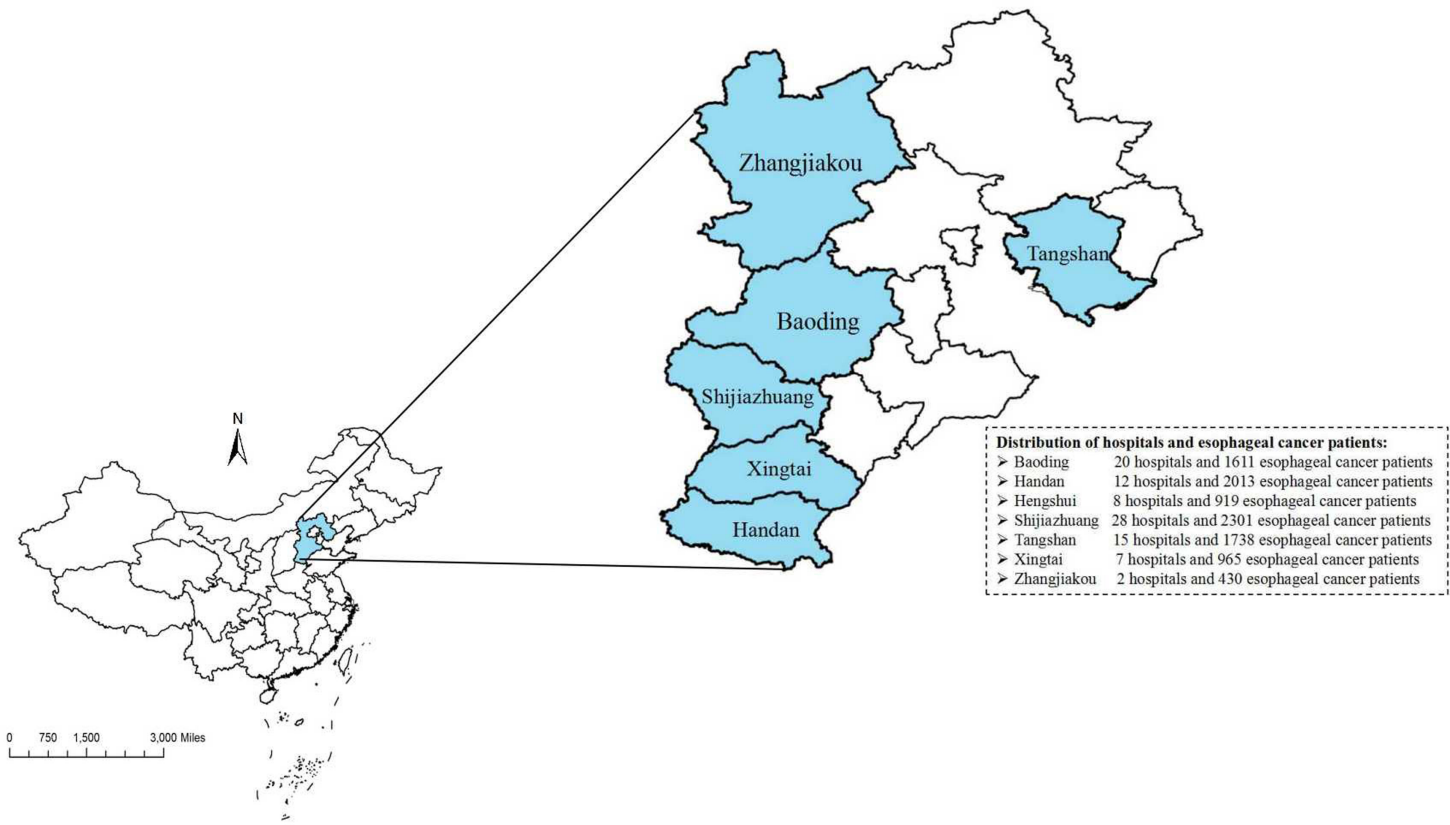
The distribution of participanting hospitals and patiens in Hebei. (ArcGIS 10.2 for Desktop software, Environmental Systems Research Institute Inc., USA, https://www.esri.com).

Data on patients were collected from 92 hospitals across 6 cities in Hebei Province, China. The distribution of participating hospitals is shown in **Figure 1**. The study included patients diagnosed with “Esophagus” according to ICD-O-3/WHO 2008, exhibiting a “Malignant” behavior code for the primary neoplasm, diagnosed between 2000 and 2020, and surgery performed in each case. Cases based solely on a death certificate or autopsy report, lacking waiting time of surgery and other clinical information or follow-up data were excluded. The implementation of various strategies for EC treatment is feasible; however, surgical intervention remains the pivotal first-line modality. Therefore, our study exclusively included patients who underwent surgical resection. Additionally, all patients included in our analysis underwent cancer-directed surgery, as opposed to considering alternative therapies such as radiotherapy or chemotherapy prior to the surgical procedure.

Information includes demographic characteristics: sex, age at diagnosis, marital status at diagnosis, household income, living areas, year of diagnosis, waiting time from diagnosis to treatment, and survival months; tumor-related information: primary tumor site, differentiation, grade, radiotherapy, chemotherapy and histology.

### Definition of variables

We defined treatment delays as the number of days between the initial medical consultation for symptoms of EC and the initiation of surgical treatment. Referring to previous studies (23, 24) and considering that EC patients in high-risk areas may experience longer delays, we classified treatment delays into four groups: no delay (0 month), brief delay (1 month), moderate delay (2 months), and long delay (≥3 months). Marital status was divided into couple and single categories, which included separated, divorced, widowed, and never married individuals. Median household income per year was categorized into three groups, low level (≤50000 yuan), middle level (50000-70000 yuan) and high level (≥70000 yuan). The living areas were classified into metropolitan counties and nonmetropolitan counties. The primary site of EC was categorized as the upper third, middle third, lower third, and other sites. The histology of EC was classified into squamous cell neoplasms, adenocarcinoma, cystic/mucinous and serous neoplasms, and other group neoplasms.

### Definition of outcomes

In terms of the clinical outcome, the primary and secondary endpoints were determined as OS and CSS, respectively. OS is a well-established endpoint in oncology trials, as it provides an overall picture of the survival of the patients in the study. It is a measure of the length of time from the initiation of treatment to the time of death from any cause. On the other hand, CSS is a more specific measure of the effectiveness of the treatment, as it only takes into account the survival of patients with the cancer type being studied. This endpoint is particularly important in cases where the treatment being investigated may have an impact on overall survival.

### Statistical analysis

The chi-square test was utilized to compare the variations among different levels of each factor. The Kaplan-Meier method was employed to analyze the 3-, 5- and 10-year OS and CSS rates, as well as the median survival time. To assess differences between the survival curves, the log-rank test was conducted. Univariate and multivariable survival analyses were performed using Cox proportional hazard regression models to calculate hazard ratios (HRs) and their corresponding 95% confidential intervals (CIs). A multivariable Cox proportional hazard regression model was constructed by incorporating risk factors that showed a significance level below 0.10 in univariate analysis. The sensitivity and specificity were evaluated by employing the receiver operating characteristic (ROC) curve, specifically quantifying the area under the curve (AUC). AUC values greater than 0.6 are considered high level fits.

The analyses were conducted using R software version 4.3.1. All statistical tests followed a two-sided approach, and a significance level of P < 0.05 was considered statistically significant.

## Results

### Characteristics of patients

During the study period, a total of 13,475 patients diagnosed with EC and undergoing surgery were included in the medical system. Among them, data on waiting time from diagnosis to surgical treatment was missing for 156 patients, while other important information was missing for 3,342 patients. Specifically, a total of 2,489 individuals did not have follow-up data regarding OS or OSS outcomes. Additionally, there were 156 cases with missing sex and age information and 697 instances where essential clinical details-such as cancer site, differentiation grade, pathological stage, and histology-were not provided. The study ultimately included a total of 9,977 patients with an average age of 64.2 (64.0-64.4) during the period from 2000 to 2020. By the end of 2020, a total of 6929 (69.4%) patients had passed away, among whom 5163 (51.3%) died due to EC. The characteristics of all EC patients stratified by waiting time from diagnosis to treatment are presented in **Table 1**. Among all the patients, 46.0% experienced a brief delay prior to receiving EC treatment, while 23.8% encountered a moderate delay and 10.1% endured a long delay.

**Table 1:** Demographic and clinical characteristics of the 9977 esophageal cancer patients categorized by waiting time from diagnosis to treatment.

Characteristics	All	Waiting time from diagnosis to treatment				P-value
		No delay	Brief delay	Moderate delay	Long delay	
**Total number**	9977	1997	4595	2375	1010	
**Age, 95%CI**	64.2 (64.0-64.4)	64.4 (63.9-64.9)	63.5 (63.2-63.8)	64.8 (64.4-65.2)	65.9 (65.3-66.5)	<0.001
**Survival months, 95%CI**	52.7 (51.7-53.6)	58.5 (55.5-61.6)	53.8 (51.9-55.7)	52.1 (50.7-53.5	49.7 (47.6-51.8)	<0.001
**Sex**						0.258
Male	8298 (83.2)	1652 (82.7)	3855 (83.9)	1967 (82.8)	824 (81.6)	
Female	1679 (16.8)	345 (17.3)	740 (16.1)	408 (17.2)	186 (18.4)	
**Year of diagnosis**						0.069
2000-2010	5019 (50.3)	1048 (52.5)	2278 (49.6)	1168 (49.2)	525 (52.0)	
2011-2020	4958 (49.7)	949 (47.5)	2317 (50.4)	1207 (50.8)	485 (48.0)	
**Marital status**						<0.001
Single	3392 (34.0)	666 (33.4)	1487 (32.4)	820 (34.5)	419 (41.5)	
Couple	6585 (66.0)	1331 (66.6)	3108 (67.6)	1555 (65.5)	591 (58.5)	
**Household income**						0.936
Low level	1038 (10.4)	219 (11.0)	479 (10.4)	243 (10.2)	97 (9.6)	
Middle level	3943 (39.5)	787 (39.4)	1826 (39.7)	932 (39.2)	398 (39.4)	
High level	4996 (50.1)	991 (49.6)	2290 (49.8)	1200 (50.5)	515 (51.0)	
**Living areas**						<0.001
Counties in metropolitan areas	8515 (85.3)	1704 (85.3)	3846 (83.7)	2077 (87.5)	888 (87.9)	
Nonmetropolitan counties	1462 (14.7)	293 (14.7)	749 (16.3)	298 (12.5)	122 (12.1)	
**Primary site**						<0.001
Upper third of esophagus	214 (2.1)	52 (2.6)	84 (1.8)	53 (2.2)	25 (2.5)	
Middle third of esophagus	1172 (11.7)	234 (11.7)	531 (11.6)	271 (11.4)	136 (13.5)	
Lower third of esophagus	7458 (74.8)	1410 (70.6)	3491 (76.0)	1820 (76.6)	737 (73.0)	
Other site	1133 (11.4)	301 (15.1)	489 (10.6)	231 (9.7)	112 (11.1)	
**Differentiation**						<0.001
Highly differentiated	881 (8.8)	245 (12.3)	297 (6.5)	195 (8.2)	144 (14.3)	
Moderately differentiated	4408 (44.2)	870 (43.6)	1993 (43.4)	1066 (44.9)	479 (47.4)	
Poor differentiated	4519 (45.3)	848 (42.5)	2221 (48.3)	1076 (45.3)	374 (37.0)	
Undifferentiated	169 (1.7)	34 (1.7)	84 (1.8)	38 (1.6)	13 (1.3)	
**Stage**						<0.001
I	2863 (28.7)	689 (34.5)	972 (21.2)	680 (28.6)	522 (51.7)	
II	2903 (29.1)	509 (25.5)	1394 (30.3)	738 (31.1)	262 (25.9)	
III	3604 (36.1)	613 (30.7)	1939 (42.2)	854 (36.0)	198 (19.6)	
IV	607 (6.1)	186 (9.3)	290 (6.3)	103 (4.3)	28 (2.8)	
**Histology**						0.001
Squamous cell neoplasms	2068 (20.7)	446 (22.3)	961 (20.9)	456 (19.2)	205 (20.3)	
Adenocarcinoma	7183 (72.0)	1416 (70.9)	3264 (71.0)	1759 (74.1)	744 (73.7)	
Cystic, mucinous and serous neoplasms	532 (5.3)	85 (4.3)	277 (6.0)	130 (5.5)	40 (4.0)	
Other	194 (1.9)	50 (2.5)	93 (2.0)	30 (1.3)	21 (2.1)	
**Radiotherapy after surgery**						<0.001
Yes	5819 (58.3)	996 (49.9)	3140 (68.3)	1360 (57.3)	323 (32.0)	
No	4158 (41.7)	1001 (50.1)	1455 (31.7)	1015 (42.7)	687 (68.0)	
**Chemotherapy after surgery**						<0.001
Yes	6308 (63.2)	1104 (55.3)	3375 (73.4)	1480 (62.3)	349 (34.6)	
No	3669 (36.8)	893 (44.7)	1220 (26.6)	895 (37.7)	661 (65.4)	

95%CI, 95% confidential interval.

The female patients accounted for 16.8% (n=1,679), while the largest group consisted of males, with a total of 83.2% (n=8,298). Among male and female EC patients, only approximately one-fifth did not experience treatment delays, with percentages of 19.9% and 20.5%, respectively. The delay in cancer treatment is consistently observed among EC cancer patients who are single at diagnose, reside in nonmetropolitan areas, and have low household income.

### Survival outcomes and univariate analysis

The OS rates at 3, 5, and 10 years for all patients were 51.6% (50.6%-52.6%), 40.6% (39.6%-41.6%), and 27.4% (26.4%-28.4%), respectively. The median OS time for the investigated cases of EC was 40 months (95% CI=38.2-41.7). The CSS rates at 3, 5, and 10 years for all patients were found to be 57.7%(56.8%-58.8%), 48.8%(47.8%-49.9%) and 38.8%(37.6%-40.0%), respectively. The median CSS time for the investigated EC cases was determined to be approximately 56.0 months (95% CI=51.8-60.1). The demographic and clinical characteristics were used to categorize the subgroup of OS and CSS rate, as well as their median survival time (**Table 2** and **Supplementary Table 1**).

**Table 2:** Overall survival rate and median survival time of the 9977 esophageal cancer patients.

Characteristics	Overall survival rate (%, 95%CI)			Survival time (months, 95%CI)	P-value
	3-year	5-year	10-year		
**All patients**	51.6 (50.6-52.6)	40.6 (39.6-41.6)	27.4 (26.4-28.4)	40.0 (38.2-41.7)	
**Sex**					0.010
Male	51.0 (49.9-52.1)	39.0 (38.8-40.9)	27.0 (25.9-28.1)	38.0 (36.1-39.8)	
Female	54.4 (52.1-56.9)	44.2 (41.8-46.7)	29.7 (27.3-32.4)	40.0 (38.2-41.7)	
**Age group (years)**					<0.001
≤44	53.5 (48.0-59.5)	43.2 (37.8-49.3)	30.6 (25.1-37.2)	43.0 (32.6-53.3)	
45-54	55.3 (52.7-58.0)	44.8 (42.2-47.5)	32.5 (29.8-35.5)	47.0 (41.0-52.9)	
55-64	53.9 (52.2-55.6)	43.4 (41.7-45.2)	25.4 (23.6-27.3)	45.0 (41.4-48.5)	
65-74	52.3 (50.7-54.0)	41.5 (39.9-43.3)	19.0 (17.3-20.9)	40.0 (36.6-43.3)	
≥75	41.9 (39.6-44.4)	28.9 (26.7-31.2)	6.21 (4.79-8.05)	26.0 (23.3-28.6)	
**Year of diagnosis**					<0.001
2000-2010	46.7 (45.3-48.1)	36.4 (35.1-37.8)	24.3 (23.2-25.5)	32.0 (30.1-33.8)	
2011-2020	56.6 (55.2-58.0)	44.8 (43.4-46.3)	31.8 (29.3-34.5)	48.0 (45.1-50.8)	
**Marital status**					<0.001
Single	49.6 (47.9-51.3)	38.2 (36.5-39.9)	25.6 (23.9-27.3)	36.0 (33.4-38.5)	
Couple	52.6 (51.4-53.8)	41.8 (40.6-43.0)	28.4 (27.1-29.6)	41.0 (38.2-41.7)	
**Household income**					<0.001
Low level	49.0 (46.0-52.1)	38.8 (35.8-41.9)	24.4 (21.4-27.8)	36.0 (31.2-40.7)	
Middle level	49.5 (47.9-51.1)	38.1 (36.6-39.7)	25.7 (24.1-27.3)	36.0 (33.6-38.3)	
High level	53.8 (52.4-55.2)	42.9 (41.5-44.3)	29.4 (28.0-30.9)	44.0 (41.0-46.9)	
**Living areas**					0.253
Metropolitan counties	51.6 (50.6-52.7)	40.8 (39.7-41.9)	27.7 (26.6-28.8)	40.0 (37.9-42.0)	
Non-metropolitan counties	51.3 (48.8-53.9)	39.3 (36.9-42.0)	26.0 (23.6-28.8)	39.0 (34.9-43.1)	
**Primary site**					<0.001
Upper third of esophagus	41.4 (35.3-48.7)	34.5 (28.6-41.7)	24.6 (19.0-32.0)	28.0 (22.9-33.0)	
Middle third of esophagus	49.0 (46.2-52.0)	37.7 (34.9-40.6)	23.6 (20.9-26.6)	35.0 (30.8-39.1)	
Lower third of esophagus	52.8 (51.7-54.0)	41.6 (40.5-42.8)	28.6 (27.5-29.8)	41.0 (38.8-43.1)	
Other site	48.0 (45.1-51.0)	37.7 (34.9-40.6)	23.9 (21.3-26.9)	33.0 (28.5-37.4)	
**Differentiation**					<0.001
Highly differentiated	71.2 (68.3-74.3)	60.6 (57.4-64.0)	41.5 (37.8-45.5)	92.0 (82.2-101.7)	
Moderately differentiated	56.4 (55.0-57.9)	45.4 (44.0-47.0)	31.1 (29.5-32.7)	48.0 (44.6-51.3)	
Poor differentiated	43.2 (41.7-44.7)	32.1 (30.7-33.5)	21.3 (19.9-22.7)	28.0 (26.4-29.5)	
Undifferentiated	45.9 (38.9-54.1)	36.0 (29.3-44.1)	24.0 (17.9-32.0)	30.0 (18.5-41.4)	
**Stage**					<0.001
I	74.0 (72.4-75.6)	62.7 (60.9-64.6)	43.8 (41.7-45.9)	97.0 (90.6-103.4)	
II	52.4 (50.6-54.3)	40.4 (38.7-42.3)	26.5 (24.7-28.4)	41.0 (37.8-44.1)	
III	38.1 (36.5-39.7)	27.4 (25.9-28.9)	17.9 (16.5-19.5)	24.0 (22.7-25.3)	
IV	22.3 (19.2-25.8)	14.8 (12.2-18.0)	11.1 (8.85-14.1)	14.0 (12.6-15.3)	
**Histology**					<0.001
Squamous cell neoplasms	45.9 (43.8-48.2)	36.6 (34.6-38.9)	21.7 (19.7-23.8)	30.0 (26.9-33.0)	
Adenocarcinoma	54.5 (53.3-55.6)	42.7 (41.5-43.8)	29.5 (28.4-30.8)	44.0 (41.8-46.1)	
Cystic, mucinous and serous neoplasms	38.7 (34.7-43.0)	29.8 (26.1-34.1)	22.2 (18.6-26.4)	24.0 (21.1-26.8)	
Other	39.5 (33.2-47.1)	33.7 (27.6-41.1)	26.5 (20.6-34.1)	21.0 (15.1-26.8)	
**Radiotherapy after surgery**					<0.001
Yes	47.2 (45.9-48.5)	35.6 (34.3-36.9)	24.0 (22.7-25.3)	54.0 (49.7-58.2)	
No	39.3 (28.8-53.7)	27.8 (18.2-42.5)	20.2 (11.6-35.3)	25.0 (22.7-27.2)	
**Chemotherapy after surgery**					<0.001
Yes	58.1 (56.5-59.7)	48.6 (47.0-50.3)	32.6 (30.9-34.4)	57.0 (52.0-61.9)	
No	47.8 (46.6-49.1)	35.9 (34.7-37.1)	24.4 (23.2-25.7)	34.0 (32.4-35.5)	
**Time from diagnosis to treatment**					<0.001
No delay	58.2 (55.2-61.3)	45.6 (42.6-48.9)	24.5 (21.5-28.1)	51.0 (44.6-57.3)	
Brief delay	53.0 (51.1-55.1)	41.9 (39.9-43.9)	22.5 (20.5-24.7)	42.0 (38.3-45.6)	
Moderate delay	50.1 (48.7-51.6)	39.1 (37.7-40.6)	20.2 (18.8-21.7)	37.0 (34.5-39.4)	
Long delay	49.8 (47.6-52.0)	39.8 (37.6-42.0)	21.4 (19.3-23.9)	36.0 (32.0-39.9)	

95%CI, 95% confidential interval.

**Figure 2:**
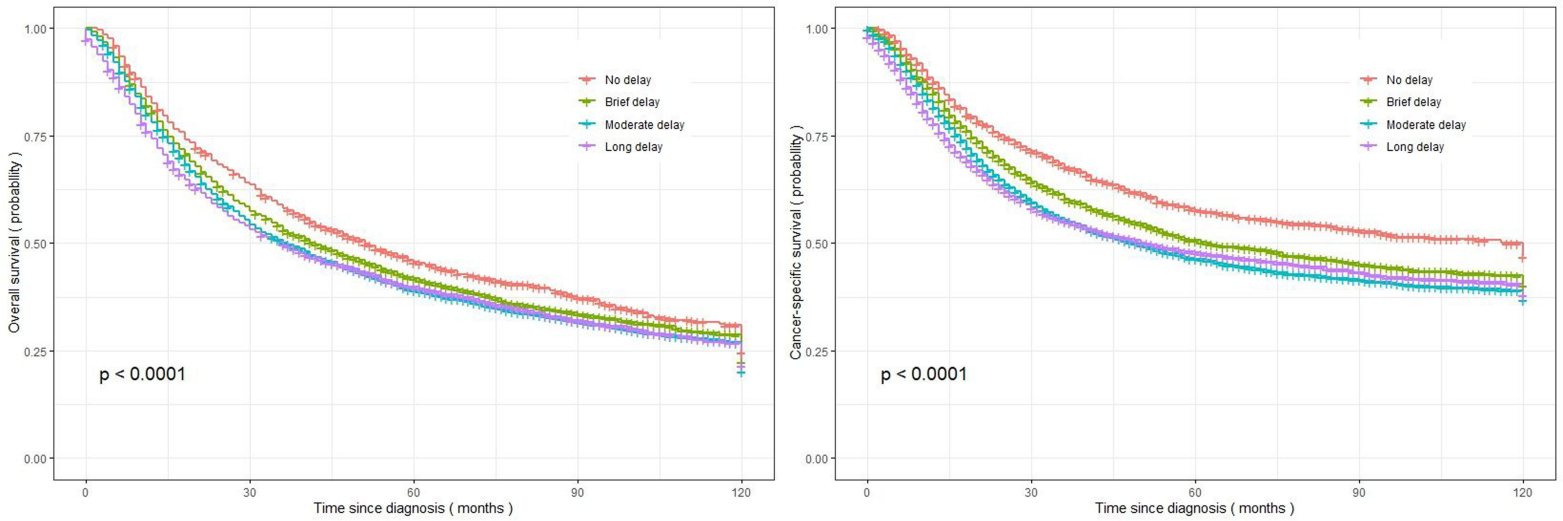
Kaplan-Meier curves representing the overall survival and cancer-specific survival of the investigated EC patients, categorized based on treatment delay (left figure: overall survival; right figure: cancer-specific survival).

Patients with EC surgery who experienced a long delay between diagnosis and treatment exhibited the lowest OS and CSS, with median survival times of 36.0 months (95% CI=32.0-39.9) and 51.0 months (95% CI=41.4-60.5), respectively. The survival rates for overall and cancer-specific outcomes at 120 months, as depicted in **Figure 2**, are based on the duration between diagnosis and treatment initiation.

The survival rates of EC in male patients, low-income patients, patients aged ≥75 and single patients were significantly lower with a 10-year OS rate of only 27%, 22.6%, 24.4%, 6.21%, and 25.6%. The 10-year OS rates of patients with lower third esophageal tumors (28.6%) and patients diagnosed with adenocarcinoma (44.0%) exhibited superior outcomes compared to those observed in other patients. The 10-year OS rate of poor/undifferentiated patients was significantly lower compared to that of other patients. The patients in stage I exhibited the longest median survival time (97.0 months), whereas those in stage IV demonstrated the shortest median survival time (14.0 months). The CSS rate showed the similar findings. The univariate analysis presented in **Table 3** and **Supplementary Table 2** **>** also corroborated these results.

**Table 3:** Univariate and multivariate analyses demonstrating the association between time from diagnosis to treatment and both overall survival and cause-specific survival in patients with esophageal cancer.

Time from diagnosis to treatment	Univariate		Multivariate	
	HR (95%CI)	*P-value*	HR (95%CI)	*P-value*
Overall survival outcome				
No delay	Reference		Reference	
Brief delay	1.11 (1.01-1.21)	<0.001	0.97 (0.89-1.07)	0.644
Moderate delay	1.18 (1.09-1.29)	<0.001	1.00 (0.91-1.09)	0.999
Long delay	1.21 (1.10-1.33)	<0.001	1.12 (1.02-1.23)	0.016
*P-value*		<0.001		0.001
Cause-specific survival outcome				
No delay	Reference		Reference	
Brief delay	1.24 (1.11-1.38)	<0.001	1.00 (0.90-1.12)	0.121
Moderate delay	1.40 (1.27-1.56)	<0.001	1.06 (0.95-1.17)	0.134
Long delay	1.42 (1.27-1.59)	<0.001	1.21 (1.08-1.36)	<0.001
*P-value*		<0.001		<0.001

HR, hazard ratio; 95%CI, 95% confidential interval.

### Multivariable analysis

The results from the Cox analyses further confirmed that sex, age, year of diagnosis race, primary site, differentiation, stage, marital status, household income, living areas and histology were significantly associated with an increased risk of all-cause and cancer-specific mortality. A long delay (≥3 months) from diagnosis to treatment was linked to a higher risk of all-cause (adjusted HR=1.12; 95%CI=1.02-1.23, p=0.001) and cancer-specific mortality (adjusted HR=1.21; 95%CI=1.08-1.36, p<0.001) compared to no delay. Patients with undifferentiated and stage IV EC demonstrated a 1.39-fold (95% CI=1.14-1.69) and 4.39-fold (95% CI=3.92-4.91) increase in the risk of all-cause mortality, respectively, when compared to those diagnosed with highly differentiated and stage I EC. Similarly, patients with undifferentiated and stage IV EC exhibited a 1.59-fold (95% CI=1.25-2.02) and 6.46-fold (95% CI=5.68-7.36) increase in the risk of cause-specific mortality, respectively, when compared to their counterparts diagnosed with highly differentiated and stage I EC (**Table 3** and **Supplementary Tables 2**, **3**).

### Subgroup analysis

We conducted subgroup analysis stratified by the demographic and clinical covariates. The observed trends suggest that most subgroups exhibit similar effects on treatment delay and OS, with patients experiencing prolonged delays being at a higher risk of mortality. The adverse impact of long delay of treatment on the survival (≥3 months) of patients with EC was consistently observed across various subgroups, including male patients (HR=1.11; 95%CI=1.00-1.23, p<0.001) and patients aged ≥75 (HR=1.20; 95%CI=1.00-1.45, p=0.010). Other risk factors associated with poor survival in cases of long treatment delay included poor differentiated (HR=1.22; 95%CI =1.06-1.41, p=0.005), stage IV EC (HR=1.61, 95%CI =1.04-2.49, p=0.001), single marital status (HR=12.5, 95%Cl=10.7-14.6, p=0.028), low level household income (HR=1.75; 95%Cl=1.26-2.43, p=0.006) and residence in nonmetropolitan counties (HR=1.43; 95%Cl=1.08-1.89, p=0.054) (**Table 4**).

**Table 4:** Subgroup analyses for the association of the association between demographic factors, clinical characteristics, and overall survival in patients with esophageal cancer.

Characteristics	Time from diagnosis to treatment				P-value
	No delay	Brief delay	Moderate delay	Long delay	
**Total number**	1997	4595	2375	1010	
Sex					
Male	Reference	0.95 (0.86-1.06)	0.96 (0.88-1.06)	1.11 (1.00-1.23)	<0.001
Female	Reference	1.12 (0.90-1.40)	1.21 (0.97-1.49)	1.09 (0.87-1.38)	0.307
Age group (years)					
≤44	Reference	0.55 (0.25-1.19)	0.69 (0.33-1.42)	0.60 (0.28-1.30)	0.419
45-54	Reference	0.98 (0.89-1.07)	0.98 (0.90-1.07)	1.08 (0.99-1.19)	0.014
55-64	Reference	1.08 (0.90-1.29)	1.01 (0.86-1.20)	1.08 (0.90-1.30)	0.494
65-74	Reference	0.95 (0.81-1.11)	0.99 (0.86-1.15)	1.15 (0.98-1.34)	0.028
≥75	Reference	1.04 (0.86-1.25)	1.08 (0.90-1.29)	1.20 (1.00-1.45)	0.010
Year of diagnosis					
2000-2010	Reference	0.98 (0.87-1.10)	1.00 (0.89-1.12)	1.14 (1.01-1.29)	0.005
2011-2020	Reference	0.97 (0.84-1.13)	1.01 (0.88-1.16)	1.05 (0.90-1.22)	0.635
Marital status					
Single	Reference	1.15 (0.99-1.33)	1.11 (0.97-1.28)	1.25 (1.07-1.46)	0.028
Couple	Reference	0.88 (0.78-0.99)	0.92 (0.83-1.03)	1.01 (0.90-1.14)	0.01
Household income					
Low level	Reference	1.44 (1.04-2.00)	1.38 (1.01-1.89)	1.75 (1.26-2.43)	0.006
Middle level	Reference	0.84 (0.73-0.97)	0.94 (0.82-1.08)	1.03 (0.89-1.19)	0.006
High level	Reference	1.02 (0.90-1.17)	0.99 (0.88-1.13)	1.08 (0.95-1.24)	0.325
Living areas					
Counties in metropolitan areas	Reference	0.96 (0.87-1.05)	0.97 (0.89-1.07)	1.07 (0.97-1.19)	0.019
Nonmetropolitan counties	Reference	1.18 (0.90-1.56)	1.28 (0.99-1.66)	1.43 (1.08-1.89)	0.054
Primary site					
Upper third of esophagus	Reference	0.90 (0.48-1.67)	0.95 (0.54-1.69)	1.07 (0.57-1.99)	0.916
Middle third of esophagus	Reference	0.85 (0.65-1.10)	0.93 (0.73-1.18)	1.13 (0.87-1.47)	0.058
Lower third of esophagus	Reference	0.98 (0.88-1.10)	1.01 (0.91-1.12)	1.10 (0.98-1.23)	0.070
Other site	Reference	1.08 (0.82-1.43)	1.05 (0.81-1.35)	1.20 (0.91-1.57)	0.428
Differentiation					
Highly differentiation	Reference	0.72 (0.53-0.98)	0.78 (0.58-1.03)	0.84 (0.62-1.13)	0.192
Moderately differentiation	Reference	1.01 (0.87-1.16)	1.03 (0.90-1.18)	1.13 (0.98-1.31)	0.147
Poor differentiation	Reference	1.04 (0.91-1.20)	1.07 (0.94-1.22)	1.22 (1.06-1.41)	0.005
Undifferentiation	Reference	0.84 (0.37-1.89)	0.84 (0.37-1.87)	1.18 (0.50-2.78)	0.632
Stage					
I	Reference	0.92 (0.79-1.08)	0.91 (0.78-1.06)	0.98 (0.83-1.15)	0.577
II	Reference	0.96 (0.81-1.14)	0.99 (0.84-1.16)	1.15 (0.96-1.37)	0.057
III	Reference	1.02 (0.85-1.21)	1.04 (0.88-1.23)	1.04 (0.87-1.25)	0.931
IV	Reference	1.09 (0.69-1.73)	1.11 (0.72-1.71)	1.61 (1.04-2.49)	0.001
Histology					
Squamous cell neoplasms	Reference	1.06 (0.87-1.30)	1.10 (0.92-1.32)	1.18 (0.96-1.44)	0.360
Adenocarcinoma	Reference	0.94 (0.84-1.05)	0.96 (0.86-1.06)	1.09 (0.98-1.22)	0.003
Cystic, mucinous and serous neoplasms	Reference	1.24 (0.80-1.94)	1.30 (0.85-1.97)	1.55 (0.97-2.48)	0.282
Other	Reference	0.90 (0.44-1.85)	0.91 (0.49-1.69)	0.79 (0.39-1.59)	0.922
Radiotherapy after surgery					
Yes	Reference	0.97 (0.86-1.10)	0.88 (0.78-0.98)	1.09 (0.96-1.23)	0.001
No	Reference	0.83 (0.61-1.13)	1.03 (0.78-1.36)	1.14 (0.85-1.52)	0.042
Chemotherapy after surgery					
Yes	Reference	0.97 (0.84-1.12)	1.01 (0.88-1.15)	1.09 (0.95-1.26)	0.087
No	Reference	0.97 (0.86-1.11)	0.92 (0.82-1.04)	1.08 (0.95-1.23)	0.054

95%CI, 95% confidential interval.

The detrimental impact of long treatment delay on the CSS was consistently observed across various subgroups, including male individuals (HR=1.18; 95%Cl=1.04-1.33, p<0.001), patients aged 65-74 (HR=1.31; 95%CI=1.08-1.59, p=0.012) and ≥75 (HR=1.33; 95%CI=1.03-1.73, p=0.057), single marital status (HR=1.38; 95%CI=1.14-1.66, p=0.004), low income (HR=1.94; 95%CI=1.32-2.86, p=0.002), residence in nonmetropolitan counties (HR=1.36; 95%CI=1.00-1.87, p=0.035), those with lower third of EC cases (HR=1.21; 95%CI=1.06-1.39, p=0.002), poor differentiated EC (HR=1.34; 95%CI=1.13-1.58, p<0.001), stage IV EC cases (HR=1.63; 95%CI=1.00-2.64, p=0.001), adenocarcinoma subtype of EC (HR=1.20; 95%CI=1.04-1.38, p<0.001) and patients who underwent surgery both before and after radiation therapy (HR=1.25; 95%CI=1.01-1.54, p=0.034) (**Supplementary Table 4**).

### Assessment of Cox proportional hazard regression models

**Figure 3:**
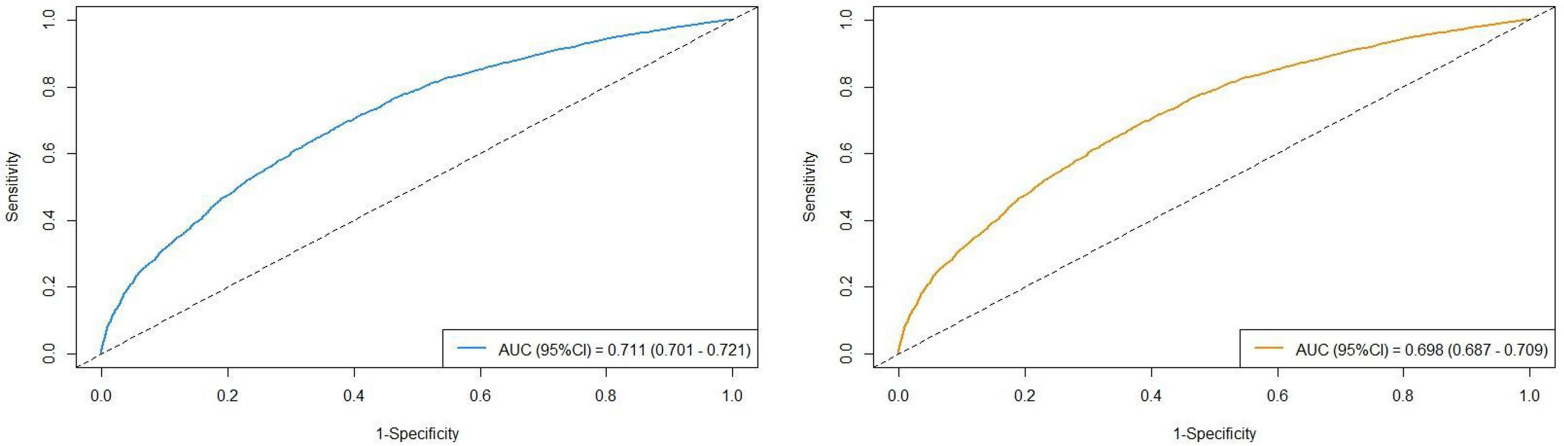
The AUC values in the cohort stratified by survival outcomes (left figure: overall survival; right figure: cancer-specific survival).

The AUC values of the three different follow-up duration are presented in **Figure 3**. The AUC values in the cohort were observed to be high: AUC_OS_ =0.711(95%CI=0.701-0.721) and AUC_CSS_ =0.698(95%CI=0.687-0.709). All model assessment results confirmed that our Cox models fit well.

## Discussion

This study investigated the impact of delayed EC treatment on OS and CSS, while controlling for individual and clinical characteristics among Chinese EC surgical patients from 2000 to 2020. The 3-, 5-, and 10-year OS rates for patients who experienced a long delay in EC treatment (≥3 months) were found to be 49.8%, 39.8%, and 21.4% respectively, while the CSS rates were determined as 55.1%, 47.8%, and 37.9%. A prolonged delay in EC treatment was independently associated with significantly higher all-cause and cancer-cause mortality among patients. The investigation into the impact of treatment delays on the long-term survival of patients with the most prevalent EC could assist physicians in developing more evidence-based interventions for this specific population.

The accumulation of substantial evidence indicates that a delay in the initiation of cancer treatment can lead to unfavorable outcomes (16–18). A comprehensive meta-analysis revealed that even a mere four-week delay in cancer treatment is associated with an elevated risk of mortality across various types of cancer, including surgical, systemic treatment, and radiotherapy indications. The findings consistently indicate a mortality risk ranging from 1.06 to 1.08 for every four-week delay in surgeries. Estimates for systemic treatment varied between hazard ratios of 1.01 and as high as 1.28, while the estimates for radiotherapy were reported as follows: radical radiotherapy for head and neck cancer (HR=1.09; 95%CI=1.05-1.14), adjuvant radiotherapy after breast conserving surgery (HR=0.98; 95%CI=0.88-1.09), and cervix cancer adjuvant radiotherapy (HR=1.23; 95%CI=1.00-1.50) (16). Similarity, a large cohort study indicated that there is a consistent association between delayed initiation of treatment and increased 5-year and 10-year predicted mortality rates across various cancers, including nonmetastatic breast, prostate, non-small cell lung, and colon cancers. This study also provided evidence suggesting that shorter time-to-treatment initiation is linked to reduced mortality rates for all examined cancer types, implying an indirect relationship between treatment deferral and mortality (21). The findings of another extensive cohort study suggested a significant prolongation in the time to treatment initiation, which is associated with an absolute increase in mortality risk ranging from 1.2% to 3.2% per week in curative settings such as early-stage breast, lung, renal, and pancreatic cancers (22). Recently conducted multi-cancer analyses revealed that, upon adjusting for confounding factors, a prolonged duration from diagnosis to treatment initiation (<6 months) was associated with adverse effects on the survival outcomes of patients diagnosed with early-stage female cancers, including non-small cell lung cancer, breast cancer, thyroid cancer, colorectal cancer, and cervical cancer (15). The relationship between the delay in EC treatment and survival outcomes has been seldom investigated. However, the impact of delayed EC treatment on survival outcomes remains understudied despite its high incidence, mortality rate, and overall poor prognosis (6).

The findings of a study conducted on 315 cases of EC in the Netherlands revealed that there were no significant differences in disease-free survival (p = 0.884) or OS (p = 0.374) between patients with waiting times of less than 8 weeks and those with waiting times of 8 weeks or more, regardless of whether they received neoadjuvant therapy followed by surgery or primary surgery with curative intent (23). Another study conducted in the Netherlands, involving 3839 cases of EC, also indicated that an extended waiting period from diagnosis to treatment for patients undergoing curative intent does not adversely impact OS (24). The former studies yielded similar findings (25, 26). In fact, a similar finding was observed in our study. We discovered that there was no significant association between surgery delay and mortality risk when the duration of surgical treatment delay was less than two months. These results may provide valuable insights for clinical practice regarding EC surgery treatment. Further analysis by Grotenhuis et al. found that a prolonged hospital delay (between endoscopic diagnosis and surgery) was associated with poorer short-term patient outcomes, characterized by higher overall morbidity and mortality rates. However, this delay did not have a detrimental effect on long-term outcomes, specifically OS (27). Our findings suggest that a delay of ≥3 months in starting EC treatment is independently associated with significantly higher rates of all-cause and cancer-specific mortality among patients. Notably, our study population was divided into four subgroups based on delay in treatment [no delay (0 month), brief delay (1 month), moderate delay (2 months), and long delay (≥3 months)], and longer delays were associated with worse OS and CSS. Specifically, 9,977 patients with EC who experienced wait-time intervals exceeding 90 days had significantly lower OS and CSS rates compared to those who received treatment within 30 days. Therefore, the use of different cutoffs for wait-time intervals may have contributed to the observed differences in OS and CSS rates. The findings of this study thus indicated that timely treatment within a clinically relevant waiting period may enhance survival outcomes, highlighting the importance of early intervention for patients.

Significantly, accumulating evidence suggested that the presence of a multi-level social network has an impact on the survival outcomes of cancer patients. The proportion of male EC patients accounted for the highest among all patients in our study, and male EC patients consistently exhibited poorer OS and CSS. This trend is not only observed in Hebei province but also globally (28). Some potential factors that might contribute to the higher incidence of EC among men could include differences in lifestyle habits, such as smoking and alcohol consumption, as well as differences in genetic susceptibility (29). The ethnic composition of the participants also demonstrated variations in previous studies. Recent studies have confirmed inconsistent survival patterns among patients with cancer from diverse ethnic and racial backgrounds (30, 31). Specifically, the survival rate of Black patients has been reported to be comparatively lower than that of Asian/Pacific Islanders. While the underlying mechanisms of survival are still under investigation, factors such as lifestyle and socioeconomic status are believed to play a pivotal role in determining survival outcomes (32, 33). To date, numerous scholars posit that the correlation between marital status and cancer survival is indicative of social support. Spouses play a pivotal role in motivating patients to seek regular medical care, thereby facilitating early diagnosis and treatment of diseases. Patients with cancer receive unwavering encouragement from their spouses to adhere to their treatment regimen and actively engage with other cancer survivors (34–36). A lot of studies have extensively explored the link between marital status and both breast cancer risk and survival (37–41). Consistent with the findings of our study, individuals from low-income backgrounds and residing in nonmetropolitan counties who experienced a prolonged delay in receiving cancer treatment demonstrated significantly improved survival outcomes compared to those who did not receive any treatment within a specific timeframe. Further investigation is required to explore the underlying factors contributing to the relationship between socioeconomic status and cancer outcomes, as well as its impact on clinical aspects. The current study revealed that patients in the long delay treatment group with poor differentiation and stage IV had significantly worse OS and CSS outcomes. These findings suggest that a prolonged time from diagnosis to treatment may negatively impact the prognosis of EC patients with poor differentiated and stage IV EC, highlighting the importance of minimizing wait-time intervals between diagnosis and treatment as a crucial prognostic factor. Notably, over an extended period marked by numerous advances in treatment paradigms and other related factors, we observed that EC patients who experienced prolonged delays in surgical intervention had poorer survival outcomes compared to those without such delays during the years 2000-2010, as opposed to the period from 2011-2020. This finding suggests that advancements in treatment paradigms may mitigate the adverse effects of surgical treatment delays to a certain extent.

To date, the bulk of the evidence underpinning the correlation between the time to treatment initiation and survival rates has been derived from data meticulously compiled in cancer registries across developed nations. However, this wealth of information may not be representative of the global cancer patient population, as it predominantly originates from high-income countries. Consequently, there is an urgent need for future research to delve into the middle- or low-income countries, where the consequences of delayed treatment are likely to be more severe due to the increased vulnerability of cancer patients to disease progression. Such an investigation would require a meticulous examination and comprehensive evaluation of the time taken from diagnosis to the initiation of treatment within various healthcare institutions. This scrutiny, when coupled with an in-depth understanding of the factors contributing to treatment delays, could have far-reaching implications. It could pave the way for public health administrators to make well-informed, tailored decisions on treatment deferrals in resource-constrained regions. Moreover, this knowledge could also facilitate the progress of global health missions, enabling healthcare providers to allocate resources more effectively and ensure that patients in need receive prompt and appropriate care. It could also serve as a vital tool in identifying gaps in the healthcare system and informing strategies to optimize treatment initiation times, ultimately improving survival rates and overall patient outcomes.

Related mechanistic studies remain limited, and the conclusions drawn are still controversial. Some studies indicated that delays in the timing of surgery or diagnosis may adversely affect cancer stage and even impact survival outcomes (42–44). Further research suggested that prolonged delays in initiating definitive treatment for EC may result in progression to advanced tumor stages, potentially adversely affecting patients’ prognosis and survival rates (45, 46). Mathematical models indicated that it may take over a decade from the emergence of the first cancer cell to the point at which a tumor becomes clinically detectable through conventional diagnostic methods. However, median potential doubling times for EC ranged from 4 to 5 days, with some cases exhibiting rapid doubling within a span of 2 to 20 days-making them among the fastest-growing tumor types (47). Furthermore, tumor growth follows an exponential pattern; therefore, even if the duration before discovery is relatively short, a significantly rapid growth rate at that point can still have substantial implications. Therefore, prolonged delays are likely detrimental factors affecting patient prognosis and survival outcomes (48).

The current study has certain limitations that warrant attention. Firstly, it is an observational study conducted retrospectively with potential selection bias, uneven baseline characteristics, and other confounding factors. Secondly, our data lacks information on patient characteristics such as lifestyle choices, educational background, insurance status, Charlson-Deyo comorbidity index score, mental health status or medical knowledge which could have influenced their prognosis, as our patient data was sourced from hospitals that did not collect individual-related information (22). Additionally, the present data does not provide detailed records regarding reasons for treatment delays which are crucial information for further investigation into this important topic and reducing cancer progression due to delayed treatment.

## Conclusion

Our findings suggest that a delay in treatment initiation for patients with EC who require surgery, lasting three months or longer, has a detrimental impact on their survival. In Hebei province, recognized as a high-risk area for EC, undergoing surgical intervention for EC within two months may yield more favorable outcomes regarding the patient’s long-term prognosis. Further investigation and validation are needed to determine if these results can serve as evidence supporting the implementation of a more comprehensive pretreatment evaluation.

## Data Availability

The raw data supporting the conclusions of this article will be made available by the authors, without undue reservation.
