# Supplementary material for: Association between surgery treatment delays and survival outcomes in patients with esophageal cancer in Hebei, China
Source: Front Oncol. 2024 Oct 28;14:1463517. doi: 10.3389/fonc.2024.1463517 (PMC11551117; doi:10.3389/fonc.2024.1463517)
Supplement: Supplementary file 1 [file Table1.docx]

**Supplementary Table 1.** Cancer-specific survival rate and median survival time of the 9977 esophageal cancer patients.

| **Characteristics** | **Cancer-specific survival rate (%, 95%CI)** | | |  |  |
| --- | --- | --- | --- | --- | --- |
|  | **3-year** | **5-year** | **10-year** | **Cancer-specific survival time**  **(months, 95%CI)** | **P-value** |
| **All patients** | 57.7(56.8-58.8) | 48.8(47.8-49.9) | 38.8(37.6-40.0) | 56.0(51.8-60.1) |  |
| **Sex** |  |  |  |  | 0.001 |
| Male | 57.0(55.9-58.1) | 48.0(46.9-49.1) | 38.2(36.9-39.5) | 53.0(48.9-57.0) |  |
| Female | 61.3(58.9-63.7) | 52.9(50.4-55.5) | 41.9(38.9-45.0) | 72.0(55.1-88.8) |  |
| **Age group (years)** |  |  |  |  | ＜0.001 |
| ≤44 | 55.8(50.4-61.9) | 46.1(40.5-52.3) | 36.3(30.6-43.0) | 48.0(33.4-62.5) |  |
| 45-54 | 59.0(56.4-61.7) | 49.8(47.1-52.6) | 43.4(40.5-46.4) | 60.0(44.1-75.8) |  |
| 55-64 | 58.5(56.8-60.3) | 49.4(47.6-51.2) | 40.2(38.3-42.3) | 58.0(50.3-65.6) |  |
| 65-74 | 58.5(56.8-60.2) | 50.2(48.4-52.0) | 37.8(35.6-40.0) | 61.0(53.1-68.8) |  |
| ≥75 | 54.0(51.5-56.7) | 44.3(41.7-47.1) | 31.9(28.6-35.6) | 46.0(39.9-52.0) |  |
| **Year of diagnosis** |  |  |  |  | ＜0.001 |
| 2000-2010 | 52.8(51.4-54.3) | 44.3(42.9-45.8) | 34.8(33.4-36.3) | 43.0(39.4-46.5) |  |
| 2011-2020 | 62.7(61.3-64.1) | 53.4(51.9-54.9) | 46.0(43.0-49.3) | 71.5(70.1-73.0) |  |
| **Marital status** |  |  |  |  | 0.089 |
| Single | 57.0(55.3-58.8) | 47.8(46.0-49.7) | 37.8(35.8-40.0) | 54.0(47.8-60.1) |  |
| Couple | 58.1(56.9-59.4) | 49.3(48.0-50.6) | 39.3(37.9-40.8) | 57.0(51.7-62.2) |  |
| **Household income** |  |  |  |  | ＜0.001 |
| Low level | 49.0(46.0-52.1) | 38.8(35.9-41.9) | 20.5(17.6-24.0) | 46.0(38.3-53.6) |  |
| Middle level | 49.5(47.9-51.1) | 38.1(36.6-39.7) | 19.8(18.3-21.4) | 50.0(44.8-55.1) |  |
| High level | 53.8(52.4-55.2) | 42.9(41.5-44.3) | 22.9(21.5-24.4) | 65.0(58.0-71.9) |  |
| **Living areas** |  |  |  |  | 0.280 |
| Metropolitan counties | 57.9(56.8-59.0) | 49.1(48.0-50.3) | 38.9(37.6-40.3) | 57.0(52.4-61.5) |  |
| Non-metropolitan counties | 56.9(54.3-59.5) | 46.9(44.2-49.7) | 38.2(35.2-41.4) | 51.0(43.0-58.9) |  |
| **Primary site** |  |  |  |  | 0.002 |
| Upper third of esophagus | 51.9(45.1-59.6) | 48.6(41.8-56.5) | 33.2(25.4-43.4) | 41.0(14.0-67.9) |  |
| Middle third of esophagus | 55.3(52.4-58.3) | 46.3(43.3-49.4) | 35.3(32.0-39.0) | 50.0(40.3-59.6) |  |
| Lower third of esophagus | 58.7(57.6-59.9) | 49.5(48.3-50.7) | 40.1(38.7-41.5) | 58.0(52.9-63.0) |  |
| Other site | 54.9(51.9-58.0) | 47.0(44.0-50.2) | 35.4(32.0-39.2) | 50.0(38.9-61.0) |  |
| **Diferentiation** |  |  |  |  | ＜0.001 |
| Highly diferentiated | 77.8(75.1-80.7) | 72.3(69.3-75.5) | 59.9(55.8-64.4) | 84.0(72.0-95.9) |  |
| Moderately diferentiated | 63.6(62.2-65.1) | 54.8(53.3-56.4) | 43.5(41.7-45.4) | 56.0(51.8-60.1) |  |
| Poor diferentiated | 48.3(46.8-49.8) | 38.5(37.0-40.0) | 30.2(28.6-31.9) | 45.0(20.8-69.1) |  |
| Undiferentiated | 525(45.2-61.0) | 45.8(38.5-54.5) | 36.7(29.4-45.8) | 34.0(31.6-36.3) |  |
| **Stage** |  |  |  |  | ＜0.001 |
| Ⅰ | 82.6(81.2-84.0) | 75.8(74.2-77.5) | 63.4(61.1-65.7) | 56.0(51.8-60.1) |  |
| Ⅱ | 58.7(56.9-60.6) | 48.6(46.7-50.5) | 36.8(34.7-39.1) | 55.0(48.7-61.2) |  |
| Ⅲ | 42.6(41.0-44.3) | 32.5(30.9-34.2) | 24.8(23.0-26.7) | 28.0(26.4-29.5) |  |
| Ⅳ | 25.8(22.4-29.7) | 18.5(15.5-22.2) | 15.4(12.5-19.0) | 15.0(13.2-16.7) |  |
| **Histology** |  |  |  |  | ＜0.001 |
| Squamous cell neoplasms | 53.1(50.9-55.4) | 46.2(43.9-48.5) | 33.7(31.1-36.6) | 47.0(39.9-54.0) |  |
| Adenocarcinoma | 60.3(59.2-61.5) | 50.7(49.5-52.0) | 41.1(39.7-42.5) | 64.0(58.1-69.8) |  |
| Cystic, mucinous and serous neoplasms | 44.1(39.9-48.7) | 35.7(31.7-40.3) | 28.4(24.2-33.3) | 28.0(23.5-32.4) |  |
| Other | 46.6(39.8-54.5) | 41.4(34.7-49.5) | 34.9(28.0-43.6) | 32.0(18.4-45.5) |  |
| **Radiotherapy after surgery** |  |  |  |  | ＜0.001 |
| Yes | 65.6(64.1-67.1) | 58.3(56.7-59.9) | 47.7(45.9-49.7) | 57.0(52.0-61.9) |  |
| No | 46.1(43.0-49.5) | 35.9(32.8-39.2) | 25.7(22.6-29.4) | 51.6(50.6-52.7) |  |
| **Chemotherapy after surgery** |  |  |  |  | ＜0.001 |
| Yes | 66.6(65.0-68.2) | 60.3(58.6-62.0) | 49.6(47.6-51.7) | 56.0(51.8-60.1) |  |
| No | 52.8(51.5-54.1) | 42.4(41.1-43.7) | 32.7(31.3-34.2) | 41.0(38.5-43.4) |  |
| **Time from diagnosis to treatment** |  |  |  |  | ＜0.001 |
| No delay | 67.7(64.8-70.8) | 57.7(54.5-61.1) | 46.9(43.1-51.1) | 63.0(53.7-72.2) |  |
| Brief delay | 60.4(58.4-62.4) | 50.7(48.5-52.9) | 40.2(37.8-42.7) | 56.0(51.8-60.1) |  |
| Moderate delay | 55.4(53.9-56.9) | 47.8(45.6-50.2) | 37.9(35.2-40.7) | 49.0(44.6-53.3) |  |
| Long delay | 55.1(52.9-57.4) | 46.4(44.9-47.9) | 36.8(35.1-38.5) | 51.0(41.4-60.5) |  |

95%CI: 95% confidential interval

**Supplementary Table 2.** Univariate and multivariate analyses demonstrating the association between demographic factors, clinical characteristics, and overall survival in patients with esophageal cancer.

| **Characteristics** | **Univariate** |  | **Multivariate** |  |
| --- | --- | --- | --- | --- |
|  | **HR(95%CI)** | **p-value** | **HR(95%CI)** | **p-value** |
| **Sex** |  | 0.011 |  | ＜0.001 |
| Male | Reference |  | Reference |  |
| Female | 0.92(0.86-0.98) |  | 0.90(0.84-0.96) |  |
| **Age group (years)** |  | ＜0.001 |  | ＜0.001 |
| ≤44 | Reference |  | Reference |  |
| 45-54 | 0.93(0.80-1.10) | 0.437 | 1.09(0.93-1.28) | 0.269 |
| 55-64 | 1.05(0.97-1.22) | 0.504 | 1.25(1.07-1.45) | 0.003 |
| 65-74 | 1.17(1.01-1.36) | 0.033 | 1.50(1.29-1.78) | ＜0.001 |
| ≥75 | 1.73(1.49-2.02) | ＜0.001 | 2.42(2.07-2.83) | ＜0.001 |
| **Year of diagnosis** |  | ＜0.001 |  | 0.124 |
| 2000-2010 | Reference |  | Reference |  |
| 2011-2020 | 0.78(0.74-0.82) |  | 0.79(0.75-1.04) |  |
| **Marital status** |  | 0.001 |  | ＜0.001 |
| Single | Reference |  | Reference |  |
| Couple | 0.91(0.87-0.96) |  | 0.87(0.83-0.92) |  |
| **Household income** |  | ＜0.001 |  | ＜0.001 |
| Low level | Reference |  | Reference |  |
| Middle level | 0.98(0.90-1.06) | 0.674 | 0.91(0.83-0.99) | 0.001 |
| High level | 0.86(0.80-0.94) | 0.001 | 0.79(0.72-0.87) | 0.003 |
| **Living areas** |  | 0.257 |  | 0.011 |
| Metropolitan counties | Reference |  | Reference |  |
| Non-metropolitan counties | 1.03(0.97-1.11) |  | 0.95(0.91-0.99) |  |
| **Primary site** |  | ＜0.001 |  | 0.001 |
| Upper third of esophagus | Reference |  | Reference |  |
| Middle third of esophagus | 0.91(0.77-1.07) | 0.279 | 0.94(0.80-1.12) | 0.532 |
| Lower third of esophagus | 0.80(0.68-0.93) | 0.006 | 0.84(0.71-0.99) | 0.040 |
| Other site | 0.92(0.77-1.09) | 0.341 | 0.95(0.80-1.13) | 0.570 |
| **Differentiation** |  | ＜0.001 |  | ＜0.001 |
| Highly differentiated | Reference |  | Reference |  |
| Moderately differentiated | 1.44(1.30-1.58) | ＜0.001 | 1.22(1.11-1.35) | ＜0.001 |
| Poor differentiated | 1.98(1.80-2.19) | ＜0.001 | 1.49(1.35-1.64) | ＜0.001 |
| Undifferentiated | 1.89(1.55-2.29) | ＜0.001 | 1.39(1.14-1.69) | 0.001 |
| **Stage** |  | ＜0.001 |  | ＜0.001 |
| Ⅰ | Reference |  | Reference |  |
| Ⅱ | 1.73(1.62-1.85) | ＜0.001 | 1.92(1.78-2.07) | ＜0.001 |
| Ⅲ | 2.43(2.28-2.59) | ＜0.001 | 3.05(2.81-3.31) | ＜0.001 |
| Ⅳ | 3.67(3.32-4.05) | ＜0.001 | 4.39(3.92-4.91) | ＜0.001 |
| **Histology** |  | ＜0.001 |  | ＜0.001 |
| Squamous cell neoplasms | Reference |  | Reference |  |
| Adenocarcinoma | 0.77(0.73-0.82) | ＜0.001 | 0.87(0.81-0.93) | ＜0.001 |
| Cystic, mucinous and serous neoplasms | 1.07(0.96-1.20) | 0.179 | 0.98(0.87-1.11) |  |
| Other | 1.02(0.86-1.22) | 0.759 | 0.95(0.80-1.14) |  |
| **Radiotherapy after surgery** |  | ＜0.001 |  | 0.602 |
| Yes | Reference |  | Reference |  |
| No | 0.54(0.43-0.67) | ＜0.001 | 0.88(0.47-1.65) | 0.707 |
| **Chemotherapy after surgery** |  | ＜0.001 |  | 0.125 |
| Yes | Reference |  | Reference |  |
| No | 0.79(0.75-0.83) | ＜0.001 | 0.83(0.79-1.07) | 0.089 |

HR: hazard ratio; 95%CI: 95% confidential interval

**Supplementary Table 3.** Univariate and multivariate analyses demonstrating the association between demographic factors, clinical characteristics, and cancer-specific survival in patients with esophageal cancer.

| **Characteristics** | **Univariate** |  | **Multivariate** |  |
| --- | --- | --- | --- | --- |
|  | **HR(95%CI)** | **p-value** | **HR(95%CI)** | **p-value** |
| **Sex** |  | 0.001 |  | ＜0.001 |
| Male | Reference |  | Reference |  |
| Female | 0.87(0.81-0.94) |  | 0.81(0.75-0.88) |  |
| **Age group (years)** |  | ＜0.001 |  | ＜0.001 |
| ≤44 | Reference |  | Reference |  |
| 45-54 | 0.86(0.73-1.02) |  | 1.04(0.87-1.23) |  |
| 55-64 | 0.90(0.77-1.05) |  | 1.10(0.94-1.29) |  |
| 65-74 | 0.92(0.79-1.08) |  | 1.24(1.06-1.46) |  |
| ≥75 | 1.13(0.96-1.33) |  | 1.69(1.43-2.00) |  |
| **Year of diagnosis** |  | ＜0.001 |  | 0.301 |
| 2000-2010 | Reference |  | Reference |  |
| 2011-2020 | 0.75(0.71-0.79) |  | 0.78(0.73-1.03) |  |
| **Marital status** |  | 0.091 |  | 0.001 |
| Single | Reference |  | Reference |  |
| Couple | 0.95(0.89-1.00) |  | 0.90(0.85-0.96) |  |
| **Household income** |  | ＜0.001 |  | ＜0.001 |
| Low level | Reference |  | Reference |  |
| Middle level | 0.97(0.88-1.06) |  | 0.89(0.81-0.99) |  |
| High level | 0.85(0.78-0.93) |  | 0.78(0.70-0.87) |  |
| **Living areas** |  | 0.283 |  | 0.018 |
| Metropolitan counties | Reference |  | Reference |  |
| Non-metropolitan counties | 1.04(0.96-1.12) |  | 0.96(0.88-0.99) |  |
| **Primary site** |  | 0.003 |  | ＜0.001 |
| Upper third of esophagus | Reference |  | Reference |  |
| Middle third of esophagus | 0.98(0.80-1.20) |  | 1.02(0.83-1.25) |  |
| Lower third of esophagus | 0.87(0.72-1.06) |  | 0.85(0.70-1.04) |  |
| Other site | 0.99(0.81-1.22) |  | 0.98(0.80-1.21) |  |
| **Differentiation** |  | ＜0.001 |  | ＜0.001 |
| Highly differentiation | Reference |  | Reference |  |
| Moderately differentiation | 1.749(1.54-1.98) |  | 1.37(1.21-1.56) |  |
| Poor differentiation | 2.70(2.38-3.06) |  | 1.78(1.57-2.03) |  |
| Undifferentiation | 2.41(1.90-3.04) |  | 1.59(1.25-2.02) |  |
| **Stage** |  | ＜0.001 |  | ＜0.001 |
| Ⅰ | Reference |  | Reference |  |
| Ⅱ | 2.39(2.19-2.60) |  | 2.60(2.35-2.86) |  |
| Ⅲ | 3.67(3.38-3.98) |  | 4.43(4.01-4.90) |  |
| Ⅳ | 5.87(5.23-6.58) |  | 6.46(5.68-7.36) |  |
| **Histology** |  |  |  | 0.058 |
| Squamous cell neoplasms | Reference |  | Reference |  |
| Adenocarcinoma | 0.81(0.76-0.86) |  | 0.95(0.87-1.03) |  |
| Cystic, mucinous and serous neoplasms | 1.22(1.08-1.38) |  | 1.09(0.96-1.25) |  |
| Other | 1.14(0.94-1.39) |  | 1.03(0.84-1.26) |  |
| **Radiotherapy after surgery** |  | ＜0.001 |  | 0.611 |
| Yes | Reference |  | Reference |  |
| No | 0.77(0.61-0.94) |  | 0.66(0.29-1.49) |  |
| **Chemotherapy after surgery** |  | ＜0.001 |  | 0.081 |
| Yes | Reference |  | Reference |  |
| No | 0.65(0.61-0.69) | ＜0.001 | 0.79(0.66-1.05) | 0.102 |

HR: hazard ratio; 95%CI: 95% confidential interval

**Supplementary Table 4.** Subgroup analyses for the association of the association between demographic factors, clinical characteristics, and cancer-specific survival in patients with esophageal cancer.

| **Characteristics** | **Time from diagnosis to treatment** | | | |  |
| --- | --- | --- | --- | --- | --- |
|  | **No delay** | **Brief delay** | **Moderate delay** | **Long delay** | **P-value** |
| **Total number** | 1997 | 4595 | 2375 | 1010 |  |
| **Sex** |  |  |  |  |  |
| Male | Reference | 0.97(0.86-1.10) | 1.00(0.89-1.12) | 1.18(1.04-1.33) | ＜0.001 |
| Female | Reference | 1.22(0.92-1.62) | 1.30(0.99-1.71) | 1.27(0.95-1.69) | 0.273 |
| **Age group (years)** |  |  |  |  |  |
| ≤44 | Reference | 0.49(0.21-1.13) | 0.61(028- 1.32) | 0.50(0.22-1.15) | 0.310 |
| 45-54 | Reference | 0.80(0.58-1.11) | 0.89(0.66-1.21) | 1.08(0.78-1.49) | 0.074 |
| 55-64 | Reference | 1.04(0.85-1.28) | 1.03(0085-1.25) | 1.14(0.92-1.41) | 0.389 |
| 65-74 | Reference | 1.06(0.87-1.28) | 1.11(0.93-1.33) | 1.31(1.08-1.59) | 0.012 |
| ≥75 | Reference | 1.05(0.81-1.35) | 1.14(0.89-1.45) | 1.33(1.03-1.73) | 0.057 |
| **Year of diagnosis** |  |  |  |  |  |
| 2000-2010 | Reference | 0.99(0.86-1.15) | 1.03(0.90-1.18) | 1.22(1.05-1.42) | 0.001 |
| 2011-2020 | Reference | 1.01(0.85-1.21) | 1.04(0.88-1.23) | 1.12(0.94-1.35) | 0.392 |
| **Marital status** |  |  |  |  |  |
| Single | Reference | 1.20(1.00-1.44) | 1.16(0.98-1.37) | 1.38(1.14-1.66) | 0.004 |
| Couple | Reference | 0.89(0.77-1.03) | 0.97(0.85-1.12) | 1.09(0.94-1.27) | 0.002 |
| **Household income** |  |  |  |  |  |
| Low level | Reference | 1.44(0.98-2.13) | 1.37(0.95-1.97) | 1.94(1.32-2.86) | 0.002 |
| Middle level | Reference | 0.83(0.70-0.98) | 0.96(0.81-1.13) | 1.10(0.92-1.31) | 0.001 |
| High level | Reference | 1.10(0.94-1.30) | 1.09(0.94-1.28) | 1.21(0.98-1.43) | 0.112 |
| **Living areas** |  |  |  |  |  |
| Counties in metropolitan areas | Reference | 0.99(0.87-1.12) | 1.05(0.93-1.17) | 1.19(0.95-1.35) | ＜0.001 |
| Nonmetropolitan counties | Reference | 1.11(0.81-1.54) | 1.12(0.83-1.51) | 1.36(1.00-1.87) | 0.035 |
| **Primary site** |  |  |  |  |  |
| Upper third of esophagus | Reference | 1.04(0.51-2.12) | 1.20(0.56-2.58) | 1.20(0.78-1.85) | 0.930 |
| Middle third of esophagus | Reference | 0.85(0.62-1.16) | 0.96(0.72-1.27) | 1.24(0.91-1.69) | 0.017 |
| Lower third of esophagus | Reference | 1.02(0.89-1.17) | 1.07(0.94-1.21) | 1.21(1.06-1.39) | 0.002 |
| Other site | Reference | 1.06(0.73-1.49) | 1.06(0.78-1.44) | 1.16(0.84-1.62) | 0.757 |
| **Diferentiation** |  |  |  |  |  |
| Highly diferentiated | Reference | 1.03(0.66-1.61) | 0.97(0.64-1.48) | 1.15(0.74-1.79) | 0.802 |
| Moderately diferentiated | Reference | 0.94(0.79-1.12) | 1.01(0.86-1.19) | 1.16(0.98-1.39) | 0.020 |
| Poor diferentiated | Reference | 1.16(0.98-1.39) | 1.13(0.96-1.32) | 1.34(1.13-1.58) | ＜0.001 |
| Undiferentiated | Reference | 0.71(0.25-1.97) | 0.85(0.31-2.31) | 1.30(0.45-3.73) | 0.408 |
| **Stage** |  |  |  |  |  |
| Ⅰ | Reference | 0.94(0.75-1.18) | 0.97(0.78-1.19) | 0.92(0.73-1.16) | 0.925 |
| Ⅱ | Reference | 0.97(0.79-1.18) | 1.01(0.84-1.21) | 1.22(0.99-1.50) | 0.019 |
| Ⅲ | Reference | 1.05(0.86-1.29) | 1.11(0.92-1.34) | 1.20(0.98-1.47) | 0.152 |
| Ⅳ | Reference | 0.96(0.58-1.60) | 1.09(0.68-1.76) | 1.63(1.00-2.64) | 0.001 |
| **Histology** |  |  |  |  |  |
| Squamous cell neoplasms | Reference | 1.04(0.82-1.32) | 1.16(0.93-1.45) | 1.23(0.97-1.56) | 0.169 |
| Adenocarcinoma | Reference | 0.97(0.85-1.11) | 0.99(0.87-1.12) | 1.20(1.04-1.38) | ＜0.001 |
| Cystic, mucinous and serous neoplasms | Reference | 1.24(0.79-1.92) | 1.38(0.91-2.08) | 1.59(1.01-2.49) | 0.168 |
| Other | Reference | 1.35(0.53-3.44) | 1.69(0.72-3.97) | 1.64(0.654.14) | 0.630 |
| **Radiotherapy after surgery** |  |  |  |  |  |
| Yes | Reference | 1.01(0.86-1.18) | 0.92(0.80-1.07) | 1.14(0.97-1.34) | 0.009 |
| No | Reference | 0.81(0.56-1.15) | 1.02(0.74-1.40) | 1.20(0.86-1.68) | 0.016 |
| **Chemotherapy after surgery** |  |  |  |  |  |
| Yes | Reference | 0.94(0.80-1.11) | 1.00(0.86-1.16) | 1.16(0.99-1.37) | 0.001 |
| No | Reference | 1.06(0.90-1.25) | 1.00(0.85-1.16) | 1.13(0.96-1.34) | 0.255 |

95%CI: 95% confidential interval
